# Professors Appointed

**Published:** 1848-10

**Authors:** 


### PROFESSORS APPOINTED.

Mr. Arnott, one of the surgeons of the Middlesex Hospital, (London) has been appointed Professor of Surgery in University College, and one of the Surgeons to University College Hospital. This appointment is announced in the London medical journals without comment. From this we infer that we were right in our remark in reference to the objections made to Professor Syme; so that although the metropolitans have gained their point in having one of themselves selected, it is doubtful whether the mass are any better pleased. One thing is certain, that they have benefited Edinburgh, and probably injured themselves, or at least their University.

In Edinburgh, Mr. Syme has been restored to his professorship in the University, and Dr. Hughes Bennett appointed to the chair of Institutes of Medicine, vacated by the election of Dr. Allen Thompson as Professor of Anatomy in the University of Glasgow.

In King's College, London, Dr. Bowman, the associate of Dr. Todd in many of his labours, has been appointed, conjointly with him, Professor of Physiology ; so that hereafter the course will be divided between these eminent physiologists.

In this country, where the number of medical Colleges is nearly equal to that of all Europe, the appointments and removals are so frequent that we do not pretend to chronicle them all. Among others, however, we have heard of the following.

Franklin Medical College, Philadelphia, Dr. Joynes, Professor of Institutes, has resigned.

Philadelphia College of Medicine,—*resigned*, Professors T. D. Mitchell, D. R. Gardiner, L. H. Beatty and J. R. Burden ; *appointed*, Drs. Savory, Kennedy, and Van Dyke.

Professor James Bryan, of Philadelphia, has been appointed Professor of Surgery in Geneva College, New York ; and

William M. McPheeters, M. D., one of the editors of the St. Louis Medical and Surgical Journal, Professor of Physiology in the St. Louis University of Mo.

